# Novel discovery of *Averrhoa bilimbi* ethanolic leaf extract in the stimulation of brown fat differentiation program in combating diet-induced obesity

**DOI:** 10.1186/s12906-019-2640-3

**Published:** 2019-09-05

**Authors:** Wai Kwan Lau, Nur Adelina Ahmad Noruddin, Abdul Hadi Ariffin, Muhd Zulkarnain Mahmud, Mohd Hasnan Mohd Noor, Azimah Amanah, Mohamad Faiz Hamzah, Zainuddin Zafarina

**Affiliations:** 1 Experimental Therapeutics Centre, Malaysian Institute of Pharmaceuticals & Nutraceuticals, Block 5-A, Halaman Bukit Gambir, 11700 Penang, Malaysia; 2 Analytical Biochemistry Research Centre, Universiti Sains Malaysia, 11800 USM, Penang, Malaysia

**Keywords:** *Averrhoa bilimbi*, Extract, Obesity, Brown adipocytes, White adipocytes

## Abstract

**Background:**

Brown adipocytes are known to promote energy expenditure and limit weight gain to combat obesity. *Averrhoa bilimbi*, locally called belimbing buluh (DBB), is mainly used as an ethnomedicine in the treatment of metabolic disorders including diabetes mellitus, hypertension and obesity. The present study aims to investigate the browning activity on white adipocytes by *A. bilimbi* leaf extract and to evaluate the potential mechanisms.

**Methods:**

Ethanolic leaf extract of *A. bilimbi* was exposed to Myf5 lineage precursor cells to stimulate adipocyte differentiation. Protein expressions of brown adipocyte markers were determined through high content screening analysis and validated through western blotting. Mito Stress Test assay was conducted to evaluate the cellular oxygen consumption rate upon *A. bilimbi* treatment.

**Results:**

*A. bilimbi* ethanolic leaf extract exhibited an adipogenesis effect similar to a PPARgamma agonist. It also demonstrated brown adipocyte differentiation in myoblastic Myf5-positive precursor cells. Expression of UCP1 and PRDM16 were induced. The basal metabolic rate and respiratory capacity of mitochondria were increased upon *A. bilimbi* treatment.

**Conclusions:**

The findings suggest that *Averrhoa bilimbi* ethanolic leaf extract induces adipocyte browning through PRDM16 activation and enhances mitochondria activity due to UCP1 up-regulation.

**Electronic supplementary material:**

The online version of this article (10.1186/s12906-019-2640-3) contains supplementary material, which is available to authorized users.

## Background

Obesity has become a major public health threat due to the sedentary lifestyle and inactivity in the society. This complex disorder warrants discovery of effective therapeutic modalities to control the epidemic, as well as its associated disorders including insulin resistance and type 2 diabetes. Fundamental to the development of obesity is an imbalance between caloric intake and energy expenditure, which leads to excessive deposition of white adipocyte depots. Brown adipocyte on the other hand plays a pivotal role in promoting energy expenditure, unlike the classic white adipocyte in fat deposition. Numerous studies showed that brown adipocyte contains large amount of mitochondria that uses cellular triglycerides and glucose as fuel for heat generation, thus improves the glucose metabolism in terms of insulin sensitivity, glucose disposal, triglyceride clearance as well as energy expenditure [1].

Despite the two main types of adipocytes, brown adipocytes can be further classified into two forms, i.e., the classical brown adipocytes, and the inducible brown adipocytes which is also termed as brite adipocytes [2]. The two forms of brown adipocytes have distinct developmental origins. Classical brown adipocyte is mainly found in the interscapular and perirenal regions and developed from myoblastic-like Myf5-positive precursors. On the other hand, inducible brown adipocyte (bride adipocytes) was suggested to arise from a non-Myf5 cell lineage and mainly appear sporadically in white adipocytes that has been exposed to chronic cold or beta-adrenergic agonists [3]. Although possessing separate origins, these two brown adipocytes have many biochemical and morphological characteristics in common such as producing multilocular lipid droplets. Enhancing the volume and activity of brown adipocyte may serve as a plausible therapeutic option to manage obesity, however, is challenging due to the low abundance in adults, especially among overweight and obese individual.

Natural products such as chili pepper and ginger were reported to promote the process of thermogenesis, inhibited adipogenesis, as well as induced brown-like phenotype in white adipocytes [4, 5]. However these spices are not recommended to be consumed in large amount due to the spicy taste and burning sensation. *Averrhoa bilimbi*, commonly known as bilimbi, is a fruit bearing tree widely found in countries of tropical Asia including Malaysia, Philippines, Indonesia and India [6]. Many health and medicinal benefits have been claimed for this plant [7]. Its leaf was reported to possess hypotriglycerimic properties by a research group in Singapore [8]. Due to the easy accessibility and traditional knowledge, the brown adipogenesis properties of this plant were studied and its molecular mechanism in adipocyte browning was evaluated.

## Methods

### Plant extractions

Leaves of *A. bilimbi*, locally known as daun belimbing buluh (DBB) were sampled from an orchard with prior permission of the owner. The botanical authentication of the specimen was conducted by Dr. Rahmad Zakaria, a botanist of Universiti Sains Malaysia and a voucher specimen 11,738 was deposited in the herbarium of Universiti Sains Malaysia, Penang, Malaysia. The 200 g of dried leaves were ground to a fine powder using dry grinder and the powder was soaked in 2 L ethanol for 3 days at room temperature. After filtration using Whatman No. 1 filter paper, the solvent was removed under reduced pressure at 45 °C using rotary evaporator to obtain 16 g crude ethanolic extract. The extract was kept in − 20 °C until further use.

### Cell culture and cytotoxicity study

3 T3-L1 preadipocytes and C2C12 myoblasts were purchased from the American Type Culture Collection (Manassas, VA, USA). They were maintained in Dulbecco’s modified Eagle’s medium (DMEM) supplemented with 10% bovine serum and 10% fetal bovine serum (FBS), respectively. Resazurin-based assay was used to determine the cytotoxicity of the extract. Both cell types were treated with DBB for 3, 6, and 9 days at concentrations range from 6.25–200 μg/ml. Cell viability was assessed by the reduction of resazurin, a non-fluorescent, blue-coloured soluble dye to a highly fluorescent pink resorufin in response to the population of cell metabolism. Resorufin was measured at an excitation wavelength of 570 nm.

### Differentiation of adipocytes and myotubes

The differentiation of adipocytes was initiated 2 days after the cells reached confluence by adding 0.5 mM isobutyl methylxanthine (IBMX), 1 μM dexamethasone (DEX) and 1 μg/ml insulin to DMEM containing 10% FBS. 2 days later, the differentiation medium was replaced by DMEM supplemented with 10% FBS and 1 μg/ml insulin. The differentiated cells were stabilised in DMEM containing 10% FBS for another 2 days. C2C12 myoblasts were induced to differentiate by substituting the supplements of 10% FBS with 10% horse serum once the cells reached confluence. Fresh differentiation medium was replaced every 2 days until the formation of myotubes were completed. Both differentiation methods were performed according to the manufacturer’s instruction.

### Differentiation of adipocytes in Myf5 lineage precursor cells

C2C12 myoblasts were cultured in DMEM supplemented with 10% FBS. Brown adipocyte differentiation was initiated after the cells reached confluence by adding 0.5 mM IBMX, 1 μM DEX, 1 μg/ml insulin and 1 μM rosiglitazone (ROSI). Two days later, the differentiation medium was replaced by DMEM supplemented with 10% FBS, 1 μg/ml insulin and 1 μM ROSI. These differentiated cells were stabilised in DMEM containing 10% FBS for another 2 days until oil droplets formation was observed. The brown adipocyte differentiation was done by adopting the method of Sharma et al. [9] with slight modifications.

### Adipocytes determination by fluorescent dye staining

To visualise the presence of adipocytes, cells were washed once with PBS and fixed with 4% paraformaldehyde for 10 min. Lipid droplets produced by adipocytes were stained by Nile Red solution. The trapped Nile Red solution between lipid droplets was visualised by the IN Cell 2200 Analyzer High Content Screening System (GE Healthcare, PA, USA) at an excitation wavelength of 460 nm.

### Determination of protein expression through high content screening analysis

3 T3-L1 preadipocytes were cultured in CellCarrier-96 black tissue culture plate (Perkin Elmer) and initiated adipocyte differentiation according to the standard protocol. After 7 days of differentiation, 3 T3-L1 adipocytes were treated with DMEM and 10% FBS containing either 100 μg/ml DBB, 1 μM ROSI or 0.5% DMSO. Cell medium containing all supplemented agents and treatment were refreshed every 2–3 days and maintained for 10 days. At day 10, the treated cells were washed with PBS and fixed with 4% paraformyldehyde. The cells were then blocked with 1% BSA before overnight incubated with primary antibody at 4 °C. Primary antibodies at 1:100 dilution used were Human/Mouse PRDM16 Sheep antibody (AF6295, R & D Systems), PGC1-alpha Goat antibody (GTX89046, GeneTex) UCP1/2/3 (FL-307) rabbit antibody (sc 28,766, Santa Cruz) and Anti-FNDC5 rabbit antibody (AB131390, Abcam). Secondary antibody incubation was conducted on the following day. The treated cells were incubated with host specific secondary antibody conjugated with fluorescent dye at 1:200 dilutions for 1 h at 37 °C. Secondary antibodies used were Thermo Fisher Pierce Rb anti-sheep IgG (H + L), FITC conjugated, Thermo Scientific Pierce Rb anti-goat IgG (H + L) secondary antibody, FITC conjugate and Santa Cruz Goat anti-rabbit IgG-CFL 488. The full plate containing treated cells was then subjected to imaging using the IN Cell Analyzer High Content Screening System. The fluorescent intensity produced from the captured cell images was then quantified using the IN Cell Developer software. Data was presented as fold increase of fluorescent intensity with relative to 0.5% DMSO treated cells.

### Western blotting analysis

C2C12 myoblasts were cultured until reaching 70% confluence. Fresh culture medium was then substituted with supplemented DMEM containing serially diluted DBB extracts ranging from 50 to 200 μg/ml. Vehicle-treated samples contained 0.5% DMSO without DBB. Cells were maintained for 7 days with fresh treated medium replenished every 2–3 days. Differentiated 3 T3-L1 adipocytes were treated with DMEM and 10% FBS containing serially diluted DBB extracts ranging from 50 to 200 μg/ml. Cells were maintained for 7 days with fresh treated medium replenished every 2–3 days. Vehicle-treated samples contained 0.5% DMSO without DBB. Cellular proteins were extracted by using M-PER® Mammalian Protein Extraction Reagent (Thermo Scientific Scientific, Waltham, MA). Protein concentrations of the lysed samples were determined by the Pierce™ BCA Protein Assay Kit (Thermo Fisher Scientific). 20 μg aliquots of protein were subjected to the sodium dodecyl sulfate polyacrylamide gel electrophoresis separation. The chromatographically separated proteins were transferred to a polyvinylidine difluoride membrane using the iBlot™ Gel Transfer Device (Thermo Fisher Scientific). After the transfer, the membrane was immersed in a blocking solution containing Phosphate Buffer Saline with 0.1% Tween-20 (PBS/T) and 3% BSA for 1 hour, followed by overnight primary antibody incubation. Primary antibodies at 1:1000 dilution used were PRDM 16 Polyclonal Antibody (PA5–20872, Thermo Fisher Scientific) and β-Actin (13E5) Rabbit mAb (#4970, Cell Signaling Technology). Then, the membrane was washed 3 times with PBS/T, 5 min each time before incubated with secondary antibody in the blocking solution at room temperature for 1 hour. Secondary antibody at dilution 1:2000 used was Goat Anti-Rabbit IgG-HRP (sc 2030, Santa Cruz). The signal of bound antibody was then enhanced by membrane immersion into the Clarity™ ECL Western Blotting Substrate (Bio-Rad, Hercules, CA) for 5–10 min. The bound antibody was then detected using the ChemiDoc XRS+ imaging system (Bio-Rad) and qualified with Image Lab 5.2.1 (Bio-Rad) image analysis software.

### Mito stress test assay

The Mito Stress Test assay was conducted to determine the basal metabolic rate (BMR), mitochondrial respiratory capacity (MRC), as well as reserve respiratory capacity (RRC) of adipocytes upon DBB treatments. 3 T3-L1 preadipocytes were cultured and differentiated in the culture plate provided by the Seahorse XF Cell Mito Stress Test Kit (Agilent Technologies, Santa Clara, CA). The differentiated cells were treated with 100 μg/ml DBB for 7 days. On day 8, the Mito Stress Test assay was carried according to the manufacturer’s instructions. The Seahorse XFe24 Analyzer (Agilent Technologies, Santa Clara, CA) was used to measure mitochondrial function in cells. This assay contained four pre-weighted mitochondrial drugs: oligomycin (oligo), carbonyl cyanide 4-(trifluoromethoxy) phenylhydrozone (FCCP), rotenone and antimycin A (rot/AA). The machine measured the baseline cellular oxygen consumption and determined the basal oxygen consumption rate (OCR). After OCR was determined, oligomycin was injected to inhibit ATP synthase to cause a decrease in OCR. FCCP was then injected to increase electron flow through the electron transport chain. FCCP is an uncoupling agent that collapses the proton gradient and disrupts the mitochondrial membrane potential to cause maximal respiration. RRC was then calculated by taking the difference between maximal respiration and basal respiration, as depicted in the manufacturer’s profile (Fig. 5a). RRC measured the ability of the cell to respond to increased energy demand. The last injection was a combination of antimycin A and rotenone to shut down mitochondrial respiration.

### LC/MS analysis

The separation of compounds was performed with an Agilent Technologies 1290 Infinity LC-system equipped with a quaternary pump (G4204A), an auto sampler, HiP Sampler (G4226A), a column heater (G1316C). Instrument control and data analysis was carried out using Agilent MassHunter Workstation software version B.06. The chromatographic separation was performed using a Zorbax Eclipse Plus C_18_ analytical column (Rapid Resolution HD, 2.1 X 50 mm, 1.8 μm) at 30 °C. The mobile phase consisted of methanol (Solvent B) and water with formic acid as solvent A (0.1 ml / 100 ml water). The flow rate was kept at 0.2 ml/min. The gradient elution started with 5%B - 95%B 0–10 min, 95%B 10–11 min, 95%B- 5%B 11–12 min and maintain 5%B for 12–15 min. The injection volume was 2 μl. Mass spectrometric analysis was performed on 6540UHD Accurate Mass Q-TOF LC/MS (G6540B). Mass spectra data was recorded on an ionization mode for a mass range of m/z 100–1700. Other mass spectrometer conditions were as follow: nebulizing gas pressure: 35 psi; drying gas flow: 8 L/min; drying gas temperature: 300 °C. The specific negative ionisation modes (m/z [M-H]^−^) were used to analyse the compounds.

## Results

### Differentiation of adipocytes in Myf5 lineage precursor cells

**Fig. 1:**
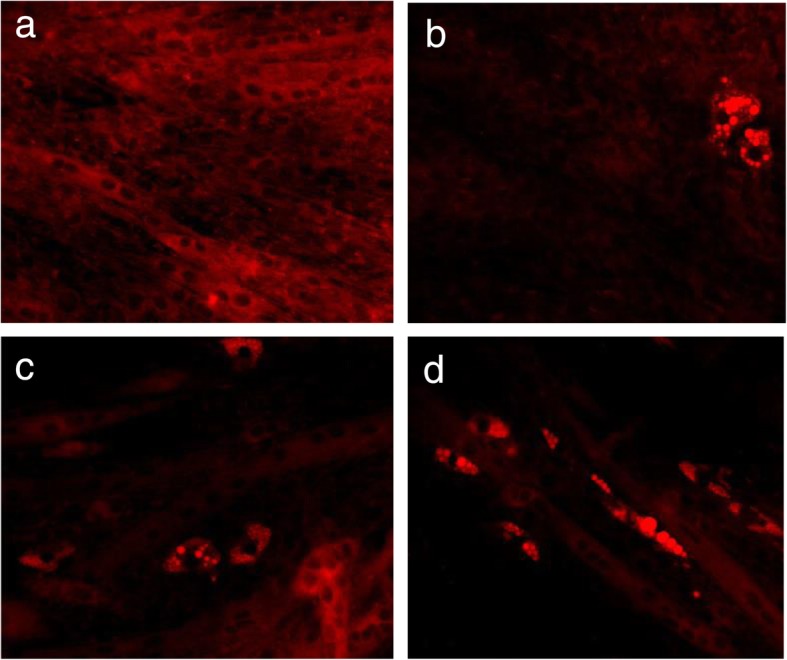
Differentiation of Myf5 lineage precursor cells upon stimulants. Lipid droplets formation upon adipogenesis was stain by Nile Red solution. Treatment of myoblasts with adipogenesis stimulants facilitated myocytes and adipocytes co-differentiation in the presence of (**b**) 1 μM ROSI, (**c**) 100 μg/ml DBB, and (**d**) 200 μg/ml DBB. (**a**) 0.5% DMSO (vehicle) showed a complete process of myogenesis without ROSI and DBB stimulations

The C2C12 cell line was used as a model system to investigate the effects of bilimbi leaf extract (DBB) on brown adipogenesis in this study. C2C12 is a murine myoblast which expressed Myf5, a myogenesis regulating protein during proliferation and skeletal muscle development. It is readily differentiated into mature myotubes in pro-myogenic medium culture, or developed into brown adipocytes upon appropriate stimulations. Rosiglitazone (ROSI), a PPARgamma agonist with known browning activities on both classic white adipocytes and inducible brown adipocytes, was served as the positive control. Adipogenesis was observed on day 4 after the initiation of differentiation (Additional file 1) by ROSI, similar phenomenon was found in DBB treated cells. Both treatments developed into fully mature adipocytes after 7 days of incubation. The development of adipocytes increased the intracellular accumulation of lipid droplets. These lipid droplets were then captured by Nile Red staining and imaged at 10X magnification (Fig. 1). Based on the cell morphology and lipid droplets accumulation, 1 μM ROSI, 100 μg/ml & 200 μg/ml DBB were demonstrated to induce adipocyte differentiation in C2C12 myoblasts. Myotubes development was also observed in DBB treated cells. Interestingly, these myoblasts were not found to undergo adipocyte differentiation in the presence of adipogenesis supplementation without DBB or ROSI stimulant. Nile Red staining did not capture lipid droplets in DMSO treated cells and was fully developed into myotubes.

**Fig. 2:**
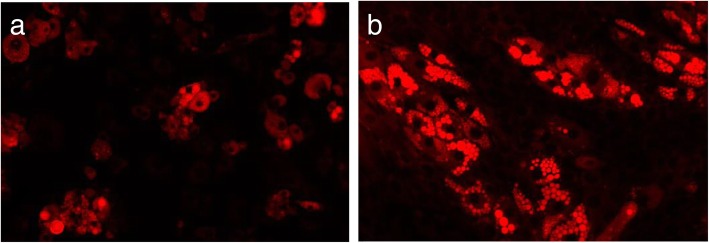
Comparison of lipid droplets formation between (**a**) Myf5 negative and (**b**) positive progenitor cells, i.e. the 3 T3-L1 adipocytes and C2C12 myocytes respectively. Lipid droplets formation upon adipogenesis was stained by Nile Red solution and imaged at 20X magnification

Brown and brite adipocyte developments were anticipated upon DBB stimulation on both Myf5-positive and -negative progenitor cells. However, it was relatively difficult to identify between white, brown and beige adipocytes from the morphological appearance (Fig. 2). Further analysis was required to support the phenotypic results.

### Protein expression determination through high content screening analysis cells

**Fig. 3:**
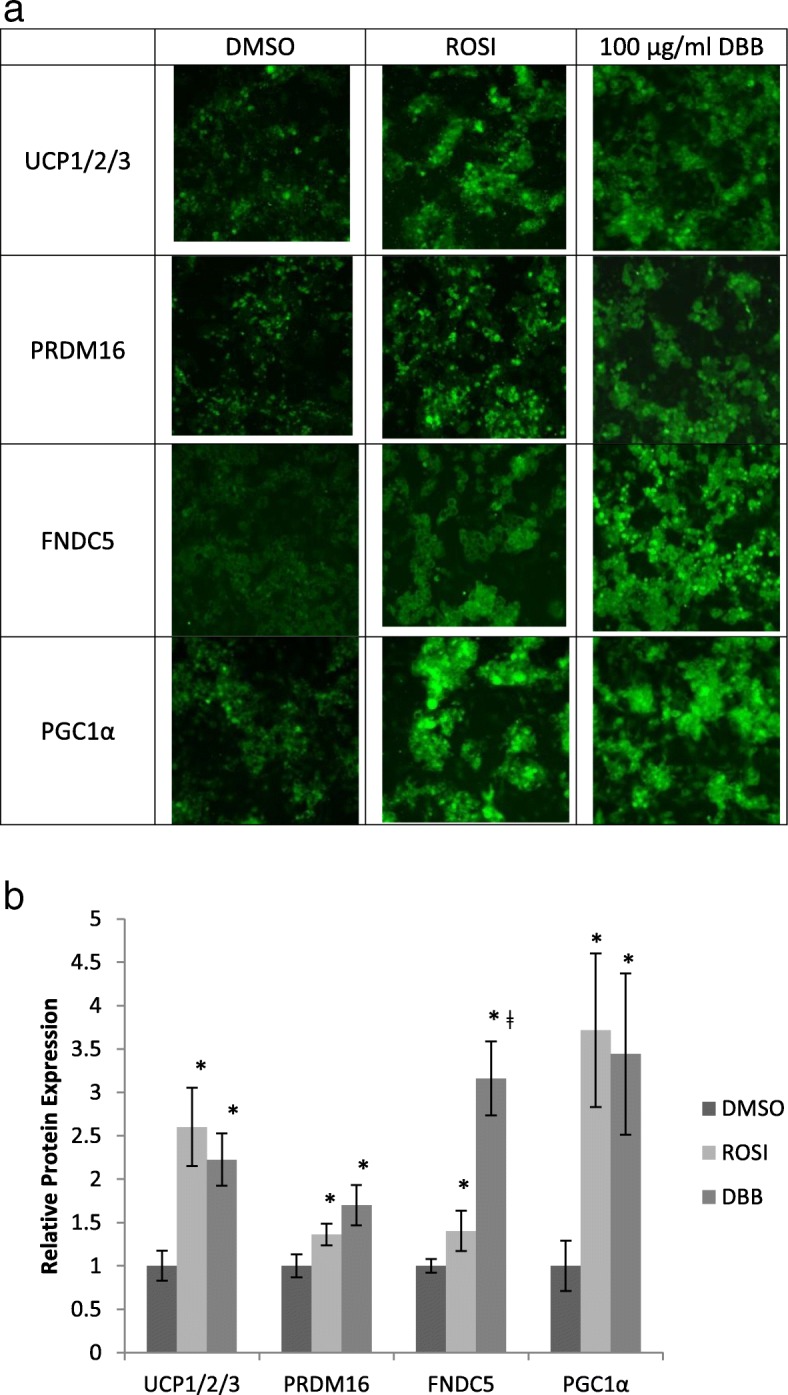
3 T3-L1 adipocytes expressed browning markers after ROSI and DBB treatments. **a** Representation of 3 T3-L1 adipocytes images acquired using IN CELL 2200 Analyzer after 10 days of treatment with either 0.5% DMSO, 1 μM ROSI or 100 μg/ml DBB, followed by staining with primary antibody for specific protein and FITC-conjugated secondary antibody respectively. **b** Quantification of the relative proteins expressions as interpreted by the sum of fluorescent signals generated from the images of cells treated with either ROSI or DBB relative to treatment with DMSO (vehicle). * denotes statistically significance (ANOVA *P* < 0.05) as compared to the vehicle. ǂ denotes statistically significance (ANOVA *P* < 0.05) between ROSI and DBB for FNDC5 expression

To anticipate the development of brite adipocytes upon stimulant, 3 T3-L1 preadipocyte was selected for this study. 3 T3-L1 is a non-Myf5 progenitor cell which only differentiates into white adipocyte upon maturity. Once fully differentiated white adipocytes were obtained, they were treated with either ROSI or DBB. These stimulants up-regulated the protein expressions of UCP1, PRDM16, FNDC5 and PGC1α in compared to DMSO treated cells, as shown in the fluorescent intensity signals captured by the IN Cell Analyzer image acquisition software (Fig. 3a). The captured intensities were then translated to quantifiable numbers. With DMSO treated cells assigned to the value of 1, signals produced from either ROSI or DBB treatments were normalised to this value (as fold increase in expression). As demonstrated in Fig. 3b, treatment with ROSI resulted in 2.6-fold increase in UCP1, 1.4-fold in PRDM16, 1.4-fold in FNDC5 and 3.7-fold in PGC1α protein expressions. Similar increments in the expression of these proteins were observed in 100 μg/ml DBB treated cells. DBB treatment on fully developed white adipocytes induced the protein expression of UCP1 and PRDM16 by 2.2-fold and 1.7-fold, FNDC by 3.2-fold and PGC-1α by 3.4-fold, significantly higher than non treated adipocytes. Treatment with DBB also showed 1.8-fold higher increment in FNDC5 than ROSI.

### Dose response study confirmed the PRDM16 expression in white adipocytes

**Fig. 4:**
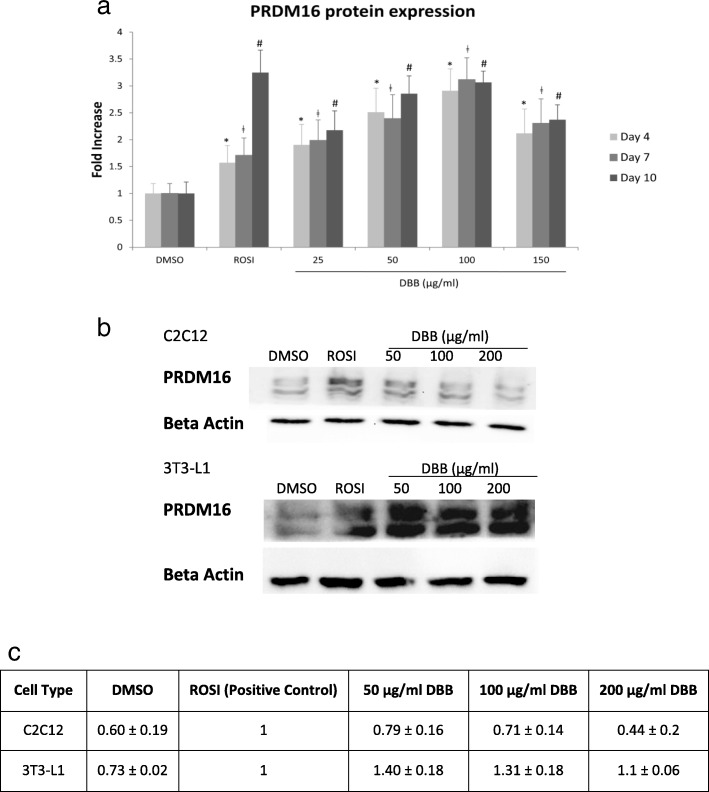
**(a)** Expression patterns of PRDM16 were responsive to treatment dose. 3 T3-L1 adipocytes were treated with either 0.5% DMSO, 1 μM ROSI or 25–150 μg/ml DBB for 4, 7 or 10 days respectively, followed by PRDM16 primary antibody labelling and FITC-conjugated secondary antibody conjugation. Image acquisition was performed using the IN Cell 2200 Analyzer and all fluorescent images were analysed using the IN Cell Developer software. * denotes statistically significance (ANOVA *P* < 0.05) as compared to DMSO (vehicle) after 4 days of treatment, ǂ denotes statistically significance (ANOVA P < 0.05) as compared to the vehicle after 7 days of treatment, and # indicates statistically significance (ANOVA P < 0.05) as compared to the vehicle after 10 days of treatment. (b) Western blot analysis was performed to detect the PRDM16 protein levels in 3 T3-L1 and C2C12 cells treated with 0.5% DMSO, 1 μM ROSI or 50–200 μg/ml DBB for 7 days. Beta actin was served as the loading control. (c) Quantification of PRDM16 protein normalised by beta actin and represented by fold expression

We then revisited the PRDM16 expression in the presence of DBB at increasing incubation period of time. PRDM16 is a known brown adipocyte determination factor which determines the thermogenic program of white adipocytes. Fully differentiated adipocytes were treated with increasing doses of DBB. Figure 4a demonstrated that treatment of 3 T3-L1 white adipocytes with ROSI could induce higher PRDM16 protein expression than the control, most remarkably observed after 7 to 10 days of treatment. Similarly, treatment with 25 μg/ml – 100 μg/ml DBB resulted in dose dependent increase in PRDM16 expression after 4, 7 and 10 days of treatments. The expression showed a decline in the 150 μg/ml DBB treatment group. The stimulation of PRDM16 expression was further validated with western blotting analysis as depicted in Fig. 4b, which detected the presence of PRDM16 in both cell types after 7 days of DBB treatment. 50 μg/ml - 100 μg/ml DBB treatments augmented PRDM16 protein expression in both cell types. A decline was observed in 200 μg/ml DBB treated cells.

### Increased in thermogenic capacity of browning process elevated cellular oxygen consumption

**Fig. 5:**
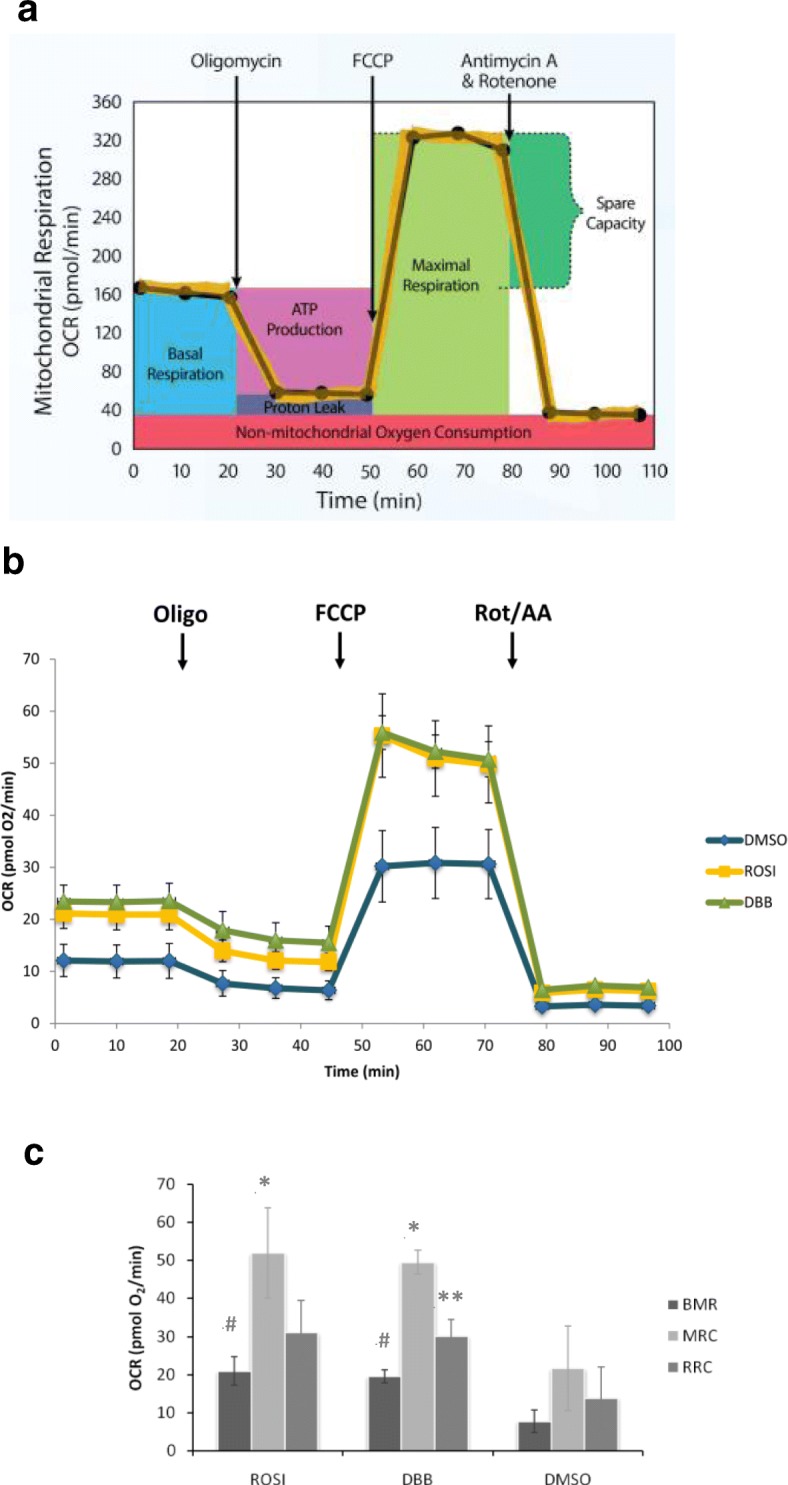
Mito Stress Test Assay was used to determine the cellular mitochondrial function. **(a)** Seahorse XF Cell Mitro Stress Test profile illustrated the key parameters of mitochondrial function namely basal respiration, ATP production, proton leak, maximal respiration, and spare respiratory capacity. **b** OCR was measured in response to consecutive addition of oligomycin (Oligo) to inhibit adenosine triphosphate (ATP) synthase, the mitochondrial uncoupler carbonyl cyanide p-triflouromethoxyphenylhydrazone (FCCP), and complex I and III inhibitors (rotenone and antimycin A (Rot/AA) respectively. **c** Treatment with DBB for 7 days markedly increased the basal OCR or basal metabolic rate (BMR), ATP-linked OCR, and maximum OCR, mitochondrial respiratory capacity (MRC), and reserve respiratory capacity (RRC). # denotes statistical significance (ANOVA P < 0.05) as compared to 0.5% DMSO for BMR, * denotes statistical significance (ANOVA P < 0.05) as compared to DMSO for MRC, and ** denotes statistical significance (ANOVA P < 0.05) as compared to DMSO for RRC

To investigate whether elevation in brown adipocyte protein expression confer metabolism and functional effects, the mitochondria activity of adipocytes upon DBB treatment was measured. Oxygen consumption rate (OCR) of treated adipocytes was determined using a Seahorse XF24 extracellular flux analyzer as demonstrated in Fig. 5b & c. Basal OCR or basal metabolic rate (BMR) was significantly higher in DBB and ROSI compared to DMSO (vehicle) treated cells, suggesting higher adenoside triphosphate (ATP) turnover and demand upon DBB and ROSI treatment. Higher basal OCR may also indicate an increase in mitochondrial mass. Three steps were involved in the measurement of mitochondrial function. In step 1, an inhibitor of mitochondrial ATP synthase, oligomycin was added immediately after the BMR stabilised. The inhibition of ATP synthase reduced electron flow through the electron transport chain. The OCR level after oligomyin injection indicated proton leak, either through non-mitochondrial respiration or mitochondria uncoupling. Higher proton leak was observed in DBB and ROSI treated cells.

The injection of proton ionophore carbonyl cyanide 4-(trifluoromethoxy) phenylhydrozone (FCCP) in step 2 induced maximal substrate oxidation and causing maximum respiration. The stimulated OCR observed was due to uncoupling of electron transfer which allowed unrestricted electron flux through the electron transport chain to achieve maximal mitochondrial respiration capacity (MRC). MRC was also higher in both DBB and ROSI treated adipocytes. In step 3, the rotenone and antimycin A mixture shut down mitochondrial respiration to measure potential non-mitochondrial respiration that was driven by processes outside the mitochondria, and to determine the oxygen consumption via electron transport chain independent mechanisms. Reserve respiratory capacity (RRC) was significantly higher in DBB treated cells, indicating the potential to increase supply of energy demand during stress or increase in cellular activities.

DBB was shown to be non-toxic in all the experiment conducted. Our findings in cytotoxicity assay also supported that DBB is non-toxic to both cell lines at a test concentration as high as 200 μg/ml after 9 days of treatment (Additional file 2).

### Liquid chromatography mass spectrometry (LC/MS) studies

**Fig. 6:**
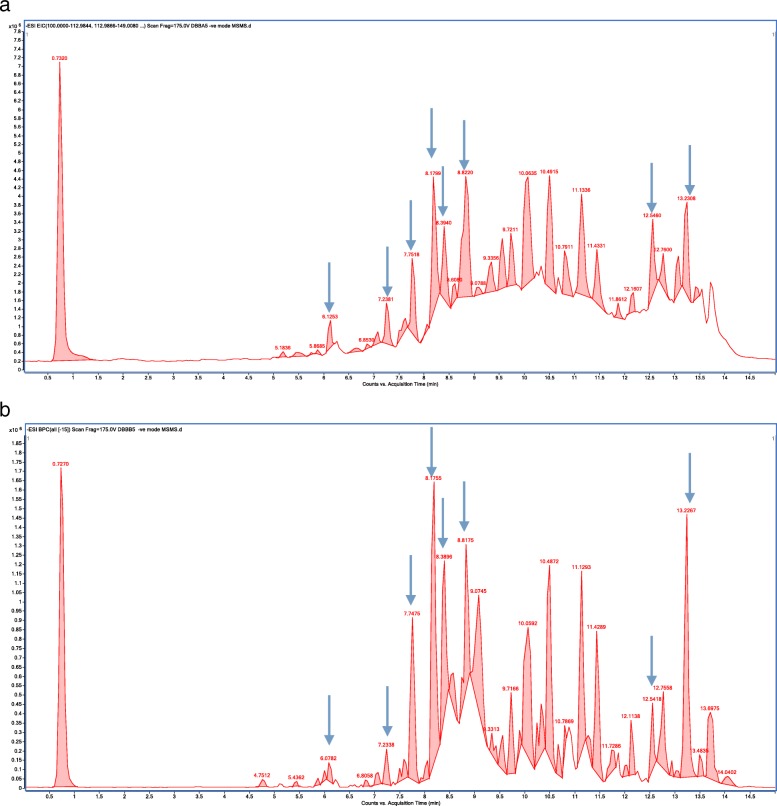
LC-MS Chromatograms of two batches of DBB extract A & B. Blue arrows indicate similar peaks obtained from the two different batches

LC/MS studies were carried out on two batches of DBB ethanolic leaf extract with chromatograms as shown in Fig. 6a & b. The LC/MS analysis has enabled us to identify 18 predominant compounds as tabulated in Table 1.

**Table 1:** Major Chemical Composition Detected in DBB Extracts using LC/MS

No.	Name	m/z	RT	Mass	Formula
1	Hallactone B	439.1084	6.1269	440.115	C20 H24 O9 S
2	Apigenin 7-allosyl-(1- > 2)-glucoside	593.1512	7.09	594.1577	C27 H30 O15
3	Kanokoside A	475.1822	7.3103	476.1892	C21 H32 O12
4	Kaempferol 3,4′-dixyloside	549.1255	7.4173	550.1317	C25 H26 O14
5	Isofurcatain 7-O-glucoside	577.1565	7.5331	578.1635	C27 H30 O14
6	Apigenin 7-cellobioside	593.1517	7.6401	594.1584	C27 H30 O15
7	(+)-Syringaresinol O-beta-D-glucoside	579.2082	7.8177	580.2152	C28 H36 O13
8	Isocytisoside	445.1134	8.0682	446.1213	C22 H22 O10
9	Vitexin 4″-O-rhamnoside	577.1566	8.1879	578.1632	C27 H30 O14
10	Retusin 7-O-neohesperidoside	591.1728	8.3377	592.1746	C28 H32 O14
11	Maysin	575.1408	8.4384	576.1478	C27 H28 O14
12	Apigenin 7-[6″-(3-Hydroxy-3-methylglutaryl)glucoside]	575.1415	8.6801	576.1483	C27 H28 O14
13	Deoxydaunorubicinol aglycone 13-O-b-glucuronide	559.1456	8.9285	560.1528	C27 H28 O13
14	3,5,6-Trimethoxy-3′,4′-methylene-dioxyfurano [2,3:7,8] flavone	395.0771	9.909	396.0845	C21 H16 O8
15	Catechin 3-O-gallate	441.0832	10.9148	442.0904	C22 H18 O10
16	Epiafzelechin 3-O-gallate	425.0875	11.3	426.0954	C22 H18 O9
17	2,4,6-Triphenyl-1-hexene	311.1817	12.7553	312.1889	C24 H24
18	5Z,9Z,12E-octadecatrienoic acid	277.2175	13.5471	278.2245	C18 H30 O2

## Discussion

*Averrhoa bilimbi*, also known as belimbing buluh by the locals, is a fast and easy growing plant. It is a long-lived species and its tree trunk can stretch up 16 to 33 ft at maturity. Its fruits are commonly consumed and have been reported to show antihypercholesterolemic activity in animal studies [10]. Malays use the juice as eye drops and regard it as a magic curative [11]. Although seldom used for oral consumption, its leaves are served as a paste on itches, swelling, rheumatism, mumps or skin eruptions and its leaf infusion is used against cough as well as after-birth tonic for babies [12]. The flower infusions are useful remedies to thrush, cold and cough [6].

Showing no sign of cytotoxicity at a concentration of 200 μg/ml, the DBB leaf extract was demonstrated to partially trigger adipocyte phenomenon in Myf5 positive cell lineage which suggested its involvement in brown adipogenesis. Rosiglitazone is a PPAR gamma agonist typically exhibiting browning effect [13]. This molecule has been known to improve glucose and lipid metabolism besides promoting transformation as well as angiogenesis of the brown adipose tissue [14]. Interestingly, DBB was found to possess similar phenotypic characteristics of a PPAR gamma agonist, and stimulating brown adipocyte differentiation in both Myf5-negative and -positive progenitor cells. This is observed when the 3 T3-L1 and C2C12 cell lines. This browning adipogenesis effect of DBB was then confirmed by browning markers elevation including UCP1, PRDM16, FNDC5 and PGC-1α. UCP1 regulates the energy balance as well as the function of brown and brite adipocytes [15]. PRDM16 is involved in the differentiation of brown adipose tissue and required at all stages of brown adipocyte tissue development [16]. FNDC5 is the precursor of irisin that promotes the white adipocytes browning by up-regulating cellular thermogenesis [17]. PGC-1α is the master regulator of mitochondrial biogenesis and interacts with multiple transcription factors through PPAR gamma [18]. PGC-1α is also a critical regulator of adaptive thermogenesis strongly induced by cold-stimulated β3-AR signalling in brown adipocytes [19]. In this study, DBB up-regulated the expression of brown adipocyte markers and suggested the occurrence of white adipocytes browning. Interestingly, FNDC5 was elevated at a higher level in DBB treated cells compared to ROSI. A more profound analysis maybe required to validate the effect of DBB on FNDC5 induced myokine irisin which represents an area for future study.

PRDM16 is the necessary activator for physiological development of brown and beige adipocytes. It stimulates brown adipogenesis in white adipocytes by stabilising the PRDM16 protein level. In the presence of PRDM16, the transcription initiation site of white adipocytes is repressed and euchromatic histone-lysine N-methyltransferase 1 (Ehmt1) is recruited to cause histone methylation [16]. Our findings showed that PRDM16 expression was augmented by DBB in a dose response manner. The up-regulating responses suggested that DBB may also play a role in the regulation of downstream browning cascades resembling the rosiglitazone [13].

Brown adipocytes rely on the functional mitochondria to convert energy into heat during adaptive thermogenesis. In brown adipocytes-mediated thermogenesis, the metabolic states are coordinated based on the mitochondrial respiration capacity [20]. The measurement of mitochondrial respiration is commonly used to assess the mitochondrial function. Higher level of respiration is usually found in brown adipocytes due to their mitochondrial abundance. Besides, the function of mitochondrion-specific metabolic processes can be appropriately determined by measuring the mitochondrial respiration.

The mechanism of DBB action in combating obesity was postulated to be related to promotion of energy expenditure and induction of mitochondria function. Mitochondria are the site of aerobic metabolism in the cells. The effectiveness of cellular metabolism is proportionated by the amount of oxygen consumed and energy produced in the mitochondria. Higher basal oxygen consumption rate (OCR) and maximum OCR measurements were observed in DBB treated cells, which reflected that DBB could contribute to higher basal metabolic rate and better respiratory capacity towards cellular metabolism.

Reserve respiratory capacity (RRC) has been a well-recognised phenomenon that correlated with enhanced cell survival [21]. The mitochondrial RRC is obtained from the difference between basal adenosine triphosphate production and its maximal activities. This RRC is regarded as the capacity available to serve the increased energy demands for maintenance of organ function, cellular repair, or detoxification of reactive species. Cells with lower RRC are more susceptible to oxidative stress whereas a larger RRC could perform better to overcome stress and maintaining optimum mitochondrial function [22]. A larger RRC was observed in DBB treated cells indicated its beneficial effect in mitochondria functions.

From the DBB treated protein expression patterns, PGC-1α was up-regulated and enhanced the activities of UCP1 which involved in mitochondrial oxidative metabolism as well as mitochondrial uncoupling. DBB also stimulated PRDM16 expression, an important factor in brown adipocytes development and stabilisation. Larger RRC was observed in the mitochondrial study with DBB. Besides, the higher proton leak in the Mito Stress Test may be contributed by DBB up-regulation of UCP1 [23].

DBB was identified to contain rich source of flavonoid compounds in LCMS analysis including apigenin derivatives, kaempferol 3,4′-dixyloside, apigenin 7-cellobioside, syringaresinol O-beta-D-glucoside, vitexin 4″-O-rhamnoside, retusin 7-O-neohesperidoside, maysin, 3,5,6-Trimethoxy-3′4’-methylene-dioxyfurano [2,3:7,8] flavone, catechin 3-O-gallate and epiafzelechin 3-O-gallate. Flavonoid compounds have been reported to reduce whole-body adiposity, ameliorate metabolic lipid disorder, improve insulin sensitivity and benefit other disorders characterised by insulin resistance in high fat diet induced obesity mice [24] . The increase energy expenditure potential of kaempferol, catechin, apigenin and other flavonoid derivatives was also suggested from the increased expressions of UCP1, PRDM16 and PGC1α, as well as their complexes regulation in energy metabolism, AMPK pathway activation, and mitochondria biogenesis mediation [25].

## Conclusions

In conclusion, our data revealed that DBB activates the browning program by up-regulating the expression of browning markers. The extracellular flux analysis demonstrated that DBB influences cellular metabolism and mitochondrial function by the expressions of PGC-1α, UCP1 and PRDM16. DBB also possesses the biological characteristics of a PPARgamma agonist similar to rosiglitazone.

## Additional files

Additional file 1: Cell Pictures Showing Adipoblast Differentiation on Day 4 treated with (a) DMSO (b) ROSI and (c) 100 μg/ml DBB. (DOCX 7840 kb)

Additional file 2: Results of DBB Cytotoxicity Study on C2C12 and 3T3-L1 Cell Lines. (A) Percentage of Viable C2C12 Cells Treated with Increased Concentrations of DBB Extract. (B) Fluorescent Intensity of C2C12 Cells After Treated with Various DBB Concentrations in μg/ml for 3, 6, and 9 Days. (C) Percentage of Viable 3T3-L1 Cells Treated with Increased Concentrations of DBB Extract. (D) Fluorescent Intensity of 3T3-L1 Cells After Treated with Various DBB Concentrations in μg/ml for 3, 6, and 9 Days. (DOCX 28 kb)

## Data Availability

The datasets used and/or analysed during the current study available from the corresponding author on reasonable request.

## Abbreviations

ATP: adenoside triphosphate
BMR: basal metabolic rate
BSA: bovine serum albumin
DBB: *Averrhoa bilimbi*
DEX: dexamethasone
DMEM: Dulbecco’s modified Eagle’s medium
DMSO: dimethyl sulfoxide
FBS: fetal bovine serum
FCCP: carbonyl cyanide 4-(trifluoromethoxy) phenylhydrozone
IBMX: isobutyl methylxanthine
MRC: mitochondrial respiratory capacity
OCR: oxygen consumption rate
PBS/T: phosphate buffer saline with 0.1% Tween-20
ROSI: rosiglitazone
rot/AA: rotenone and antimycin A
RRC: reserve respiratory capacity
