# Supplementary material for: Novel discovery of Averrhoa bilimbi ethanolic leaf extract in the stimulation of brown fat differentiation program in combating diet-induced obesity
Source: BMC Complement Altern Med. 2019 Sep 5;19:243. doi: 10.1186/s12906-019-2640-3 (PMC6727514; doi:10.1186/s12906-019-2640-3)
Supplement: Supplementary file 1 — Cell Pictures Showing Adipoblast Differentiation on Day 4 treated with (a) DMSO (b) ROSI and (c) 100 μg/ml DBB. (DOCX 7840 kb) [file 12906_2019_2640_MOESM1_ESM.docx]

**Supplementary Materials**

**Additional File 1.**

1. **DMSO treated

**
2. **ROSI treated

**
3. **100 µg/ml DBB treated**

**

**
