# Supplementary material for: Novel discovery of Averrhoa bilimbi ethanolic leaf extract in the stimulation of brown fat differentiation program in combating diet-induced obesity
Source: BMC Complement Altern Med. 2019 Sep 5;19:243. doi: 10.1186/s12906-019-2640-3 (PMC6727514; doi:10.1186/s12906-019-2640-3)
Supplement: Supplementary file 2 — Results of DBB Cytotoxicity Study on C2C12 and 3T3-L1 Cell Lines. (A) Percentage of Viable C2C12 Cells Treated with Increased Concentrations of DBB Extract. (B) Fluorescent Intensity of C2C12 Cells After Treated with Various DBB Concentrations in μg/ml for 3, 6, and 9 Days. (C) Percentage of Viable 3T3-L1 Cells Treated with Increased Concentrations of DBB Extract. (D) Fluorescent Intensity of 3T3-L1 Cells After Treated with Various DBB Concentrations in μg/ml for 3, 6, and 9 Days. (DOCX 28 kb) [file 12906_2019_2640_MOESM2_ESM.docx]

**Additional File 2.**

**(A)**

**(B)**

**(C)**

**(D)**
